# Guidelines for clinical trial protocols for interventions involving artificial intelligence: the SPIRIT-AI Extension

**DOI:** 10.1136/bmj.m3210

**Published:** 2020-09-09

**Authors:** Samantha Cruz Rivera, Xiaoxuan Liu, An-Wen Chan, Alastair K Denniston, Melanie J Calvert, SPIRIT-AI and CONSORT-AI Working Group, Hutan Ashrafian, Andrew L Beam, Gary S Collins, Ara Darzi, Jonathan J Deeks, M Khair ElZarrad, Cyrus Espinoza, Andre Esteva, Livia Faes, Lavinia Ferrante di Ruffano, John Fletcher, Robert Golub, Hugh Harvey, Charlotte Haug, Christopher Holmes, Adrian Jonas, Pearse A Keane, Christopher J Kelly, Aaron Y Lee, Cecilia S Lee, Elaine Manna, James Matcham, Melissa McCradden, David Moher, Joao Monteiro, Cynthia Mulrow, Luke Oakden-Rayner, Dina Paltoo, Maria Beatrice Panico, Gary Price, Samuel Rowley, Richard Savage, Rupa Sarkar, Sebastian J Vollmer, Christopher Yau

**Affiliations:** 1 Centre for Patient Reported Outcome Research, Institute of Applied Health Research, University of Birmingham, Birmingham, UK.; 2 Birmingham Health Partners Centre for Regulatory Science and Innovation, University of Birmingham, Birmingham, UK; 3 Academic Unit of Ophthalmology, Institute of Inflammation and Ageing, College of Medical and Dental Sciences, University of Birmingham, UK; 4 Department of Ophthalmology, University Hospitals Birmingham NHS Foundation Trust, Birmingham, UK; 5 Moorfields Eye Hospital NHS Foundation Trust, London, UK; 6 Health Data Research UK, London, UK; 7 Department of Medicine, Women’s College Research Institute, Women’s College Hospital, University of Toronto, Ontario, Canada; 8 National Institute of Health Research Biomedical Research Centre for Ophthalmology, Moorfields Hospital London NHS Foundation Trust and University College London, Institute of Ophthalmology, London, UK; 9 National Institute of Health Research Surgical Reconstruction and Microbiology Centre, University of Birmingham and University Hospitals Birmingham NHS Foundation Trust, Birmingham, UK; 10 National Institute of Health Research Birmingham Biomedical Research Centre, University of Birmingham and University Hospitals Birmingham NHS Foundation Trust, Birmingham, UK; 11 National Institute of Health Research Applied Research Collaborative West Midlands, Birmingham, UK

## Abstract

The SPIRIT 2013 (The Standard Protocol Items: Recommendations for Interventional Trials) statement aims to improve the completeness of clinical trial protocol reporting, by providing evidence-based recommendations for the minimum set of items to be addressed. This guidance has been instrumental in promoting transparent evaluation of new interventions. More recently, there is a growing recognition that interventions involving artificial intelligence need to undergo rigorous, prospective evaluation to demonstrate their impact on health outcomes.

The SPIRIT-AI extension is a new reporting guideline for clinical trials protocols evaluating interventions with an AI component. It was developed in parallel with its companion statement for trial reports: CONSORT-AI. Both guidelines were developed using a staged consensus process, involving a literature review and expert consultation to generate 26 candidate items, which were consulted on by an international multi-stakeholder group in a 2-stage Delphi survey (103 stakeholders), agreed on in a consensus meeting (31 stakeholders) and refined through a checklist pilot (34 participants).

The SPIRIT-AI extension includes 15 new items, which were considered sufficiently important for clinical trial protocols of AI interventions. These new items should be routinely reported in addition to the core SPIRIT 2013 items. SPIRIT-AI recommends that investigators provide clear descriptions of the AI intervention, including instructions and skills required for use, the setting in which the AI intervention will be integrated, considerations around the handling of input and output data, the human-AI interaction and analysis of error cases.

SPIRIT-AI will help promote transparency and completeness for clinical trial protocols for AI interventions. Its use will assist editors and peer-reviewers, as well as the general readership, to understand, interpret and critically appraise the design and risk of bias for a planned clinical trial.

## Introduction

A clinical trial protocol is an essential document produced by study investigators detailing a priori the rationale, proposed methods and plans for how a clinical trial will be conducted.^1^ ^2^ This key document is used by external reviewers (funding agencies, regulatory bodies, research ethics committees, journal editors, peer reviewers and institutional review boards, and increasingly the wider public) to understand and interpret the rationale, methodological rigor and ethical considerations of the trial. Additionally, trial protocols provide a shared reference point to support the research team in conducting a high-quality study.

Despite their importance, the quality and completeness of published trial protocols are variable.^1^ ^2^ The Standard Protocol Items: Recommendations for Interventional Trials (SPIRIT) statement was published in 2013 to provide guidance for the minimum reporting content of a clinical trial protocol and has been widely endorsed as an international standard.^3^ ^4^ ^5^ The SPIRIT statement published in 2013 provides minimum guidance applicable for all clinical trial interventions, but recognises that certain interventions may require extension or elaboration of these items.^1^ ^2^ Artificial intelligence (AI) is an area of enormous interest, with strong drivers to accelerate new interventions through to publication, implementation and market.^6^ While AI systems have been researched for some time, recent advances in deep learning and neural networks have gained significant interest for their potential in health applications. Examples of such applications of these are wide-ranging and include AI systems for screening and triage,^7^ ^8^ diagnosis,^9^ ^10^ ^11^ ^12^ prognostication,^13^ ^14^ decision-support^15^ and treatment recommendation.^16^ However, in most recent cases, the majority of published evidence consists of *in silico*, early-phase validation. It has been recognised that most recent AI studies are inadequately reported and existing reporting guidelines do not fully cover potential sources of bias specific to AI systems.^17^ The welcome emergence of randomised controlled trials (RCTs) seeking to evaluate clinical efficacy of newer interventions based on, or including, an AI component (hereafter ‘AI interventions’) 15 18 19 20 21 22 23 has similarly been met with concerns about design and reporting.^17^ ^24^ ^25^ ^26^ This has highlighted the need to provide reporting guidance that is ‘fit-for-purpose’ in this domain.

SPIRIT-AI (as part of the SPIRIT-AI and CONSORT-AI initiative) is an international initiative supported by SPIRIT and the EQUATOR (Enhancing Quality and Transparency of Health Research) Network to extend or elaborate the existing SPIRIT 2013 statement where necessary, to develop consensus-based AI-specific protocol guidance.^27^ ^28^ It is complementary to the CONSORT-AI statement, which aims to promote high quality reporting of AI trials. This article describes the methods used to identify and evaluate candidate items and gain consensus. In addition, it also provides the full SPIRIT-AI checklist including new items and their accompanying explanations.

## Methods

The SPIRIT-AI and CONSORT-AI extensions were simultaneously developed for clinical trial protocols and trial reports. An announcement for the SPIRIT-AI and CONSORT-AI initiative was published in October 2019,^27^ and the two guidelines were registered as reporting guidelines under development on the EQUATOR library of reporting guidelines in May 2019. Both guidelines were developed in accordance with the EQUATOR Network’s methodological framework.^29^ The SPIRIT-AI and CONSORT-AI steering group, consisting of 15 international experts, was formed to oversee the conduct and methodology of the study. Definitions of key terms are contained in the glossary box 1.

Box 1 **Glossary**

*Artificial intelligence (AI)*—The science of developing computer systems which can perform tasks normally requiring human intelligence.
*AI intervention*—A health intervention which relies on an artificial intelligence/machine learning component to serve its purpose.
*CONSORT*—Consolidated Standards of Reporting Trials.
*CONSORT-AI extension item*—An additional checklist item to address AI-specific content that is not adequately covered by CONSORT 2010.
*Class activation map*—Class activation maps are particularly relevant to image classification AI interventions. Class activation maps are visualisations of the pixels that had the greatest influence on predicted class, by displaying the gradient of the predicted outcome from the model with respect to the input. They are also referred to as saliency maps or heatmaps.
*Health outcome*—Measured variables in the trial which are used to assess the effects of an intervention.
*Human-AI interaction*—The process of how users/humans interact with the AI intervention, for the AI intervention to function as intended.
*Clinical outcome*—Measured variables in the trial which are used to assess the effects of an intervention.
*Delphi study*—A research method which derives the collective opinions of a group through a staged consultation of surveys, questionnaires, or interviews, with an aim to reach consensus at the end.
*Development environment*—The clinical and operational settings from which the data used for training the model is generated. This includes all aspects of the physical setting (such as geographical location, physical environment), operational setting (such as integration with an electronic record system, installation on a physical device) and clinical setting (such as primary/secondary/tertiary care, patient disease spectrum).
*Fine-tuning*—Modifications or additional training performed on the AI intervention model, done with the intention of improving its performance.
*Input data*—The data that need to be presented to the AI intervention to allow it to serve its purpose.
*Machine learning (ML)*—A field of computer science concerned with the development of models/algorithms which can solve specific tasks by learning patterns from data, rather than by following explicit rules. It is seen as an approach within the field of artificial intelligence.
*Operational environment*—The environment in which the AI intervention will be deployed, including the infrastructure required to enable the AI intervention to function.
*Output data*—The predicted outcome given by the AI intervention based on modelling of the input data. The output data can be presented in different forms, including a classification (including diagnosis, disease severity or stage, or recommendation such as referability), a probability, a class activation map, etc. The output data typically provides additional clinical information and/or triggers a clinical decision.
*Performance error*—Instances where the AI intervention fails to perform as expected. This term can describe different types of failures and it is up to the investigator to specify what should be considered a performance error, preferably based on prior evidence. This can range from small decreases in accuracy (compared to expected accuracy), to erroneous predictions, or the inability to produce an output in certain cases.
*SPIRIT*—Standard Protocol Items: Recommendations for Interventional Trials.
*SPIRIT-AI*—An additional checklist item to address AI-specific content that is not adequately covered by SPIRIT 2013.
*SPIRIT-AI elaboration item*—Additional considerations to an existing SPIRIT 2013 item when applied to AI interventions.

### Ethical approval

This study was approved by the ethical review committee at the University of Birmingham, UK (ERN_19-1100). Participant information was provided to Delphi participants electronically before survey completion and before the consensus meeting. Delphi participants provided electronic informed consent, and written consent was obtained from consensus meeting participants.

### Literature review and candidate item generation

An initial list of candidate items for the SPIRIT-AI and CONSORT-AI checklists was generated through review of the published literature and consultation with the steering group and known international experts. A search was performed on 13 May 2019 using the terms “artificial intelligence,” “machine learning,” and “deep learning” to identify existing clinical trials for AI interventions listed within the US National Library of Medicine’s clinical trial registry, ClinicalTrials.gov. There were 316 registered trials on ClinicalTrials.gov, of which 62 were completed and seven had published results.^22^ ^30^ ^31^ ^32^ ^33^ ^34^ ^35^ Two studies were reported with reference to the CONSORT statement,^22^ ^34^ and one study provided an unpublished trial protocol.^34^ The Operations Team (XL, SCR, MJC, and AKD) identified AI-specific considerations from these studies and reframed them as candidate reporting items. The candidate items were also informed by findings from a previous systematic review which evaluated the diagnostic accuracy of deep learning systems for medical imaging.^17^ After consultation with the steering group and additional international experts (n=19), 29 candidate items were generated: 26 of which were relevant for both SPIRIT-AI and CONSORT-AI and three of which were relevant only for CONSORT-AI. The Operations Team mapped these items to the corresponding SPIRIT and CONSORT items, revising the wording and providing explanatory text as required to contextualise the items. These items were included in subsequent Delphi surveys.

### Delphi consensus process

In September 2019, 169 key international experts were invited to participate in the online Delphi survey to vote on the candidate items and suggest additional items. Experts were identified and contacted via the steering group and were allowed one round of snowball recruitment, where contacted experts could suggest additional experts. In addition, individuals who made contact following publication of the announcement were included.^27^ The steering group agreed that individuals with expertise in clinical trials and AI/ML, as well as key users of the technology should be well represented in the consultation. Stakeholders included healthcare professionals, methodologists, statisticians, computer scientists, industry representatives, journal editors, policy makers, health informaticists, law and ethicists, regulators, patients, and funders. Participant characteristics are described in the appendix (page 2: supplementary table 1). Two online Delphi surveys were conducted. DelphiManager software (version 4.0), developed and maintained by the COMET (Core Outcome Measures in Effectiveness Trials) initiative, was used to undertake the e-Delphi surveys. Participants were given written information about the study and asked to provide their level of expertise within the fields of (i) AI/ML, and (ii) clinical trials. Each item was presented for consideration (26 for SPIRIT-AI and 29 for CONSORT-AI). Participants were asked to vote on each item using a 9-point scale: (1-3) not important, (4-6) important but not critical, and (7-9) important and critical. Respondents provided separate ratings for SPIRIT-AI and CONSORT-AI. There was an option to opt out of voting for each item, and each item included space for free text comments. At the end of the Delphi survey, participants had the opportunity to suggest new items. One hundred and three responses were received for the first Delphi round, and 91 (88% of participants from round one) responses received for the second round. The results of the Delphi surveys informed the subsequent international consensus meeting. Twelve new items were proposed by the Delphi study participants and were added for discussion at the consensus meeting. Data collected during the Delphi survey were anonymised and item-level results were presented at the consensus meeting for discussion and voting.

The two-day consensus meeting took place in January 2020 and was hosted by the University of Birmingham, UK, to seek consensus on the content of SPIRIT-AI and CONSORT-AI. Thirty one international stakeholders were invited from the Delphi survey participants to discuss the items and vote for their inclusion. Participants were selected to achieve adequate representation from all the stakeholder groups. Thirty eight items were discussed in turn, comprising the 26 items generated in the initial literature review and item generation phase (these 26 items were relevant to both SPIRIT-AI and CONSORT-AI; 3 extra items relevant to CONSORT-AI only were also discussed) and the 12 new items proposed by participants during the Delphi surveys. Each item was presented to the consensus group, alongside its score from the Delphi exercise (median and interquartile ranges) and any comments made by Delphi participants related to that item. Consensus meeting participants were invited to comment on the importance of each item and whether the item should be included in the AI extension. In addition, participants were invited to comment on the wording of the explanatory text accompanying each item and the position of each item relative to the SPIRIT 2013 and CONSORT 2010 checklists. After open discussion of each item and the option to adjust wording, an electronic vote took place with the option to include or exclude the item. An 80% threshold for inclusion was pre-specified and deemed reasonable by the steering group to demonstrate majority consensus. Each stakeholder voted anonymously using Turning Point voting pads (Turning Technologies LLC, Ohio, USA; version 8.7.2.14).

### Checklist pilot

Following the consensus meeting, attendees were given the opportunity to make final comments on the wording and agree that the updated SPIRIT-AI and CONSORT-AI items reflected discussions from the meeting. The Operations Team assigned each item as extension or elaboration item based on a decision tree and produced a penultimate draft of the SPIRIT-AI and CONSORT-AI checklist (supplementary fig 1). A pilot of the penultimate checklist was conducted with 34 participants to ensure clarity of wording. Experts participating in the pilot included: a) Delphi participants who did not attend the consensus meeting and b) external experts, who had not taken part in the development process but who had reached out to the steering committee after the Delphi study commenced. Final changes were made on wording only to improve clarity for readers, by the Operations Team (supplementary fig 2).

## Results

### SPIRIT-AI checklist items and explanations

The SPIRIT-AI Extension recommends that, in conjunction with existing SPIRIT 2013 items, 15 items (12 extensions and 3 elaborations) should be addressed for trial protocols of AI-interventions. These items were considered sufficiently important for clinical trial protocols for AI interventions that should be routinely reported in addition to the core SPIRIT 2013 checklist items. Table 1 lists the SPIRIT-AI items.

**Table 1:** SPIRIT-AI checklist

Section	Item	SPIRIT 2013 Item*	SPIRIT-AI item		Addressed on page No†
**Administrative information**					
Title	1	Descriptive title identifying the study design, population, interventions, and, if applicable, trial acronym	SPIRIT-AI 1(i) Elaboration	Indicate that the intervention involves artificial intelligence/machine learning and specify the type of model.	
			SPIRIT-AI 1(ii) Elaboration	Specify the intended use of the AI intervention.	
Trial registration	2a	Trial identifier and registry name. If not yet registered, name of intended registry			
	2b	All items from the World Health Organization Trial Registration Data Set			
Protocol version	3	Date and version identifier			
Funding	4	Sources and types of financial, material, and other support			
Roles and responsibilities	5a	Names, affiliations, and roles of protocol contributors			
	5b	Name and contact information for the trial sponsor			
	5c	Role of study sponsor and funders, if any, in study design; collection, management, analysis, and interpretation of data; writing of the report; and the decision to submit the report for publication, including whether they will have ultimate authority over any of these activities			
	5d	Composition, roles, and responsibilities of the coordinating centre, steering committee, endpoint adjudication committee, data management team, and other individuals or groups overseeing the trial, if applicable (see Item 21a for data monitoring committee)			
**Introduction**					
Background and rationale	6a	Description of research question and justification for undertaking the trial, including summary of relevant studies (published and unpublished) examining benefits and harms for each intervention	SPIRIT-AI 6a (i) Extension	Explain the intended use of the AI intervention in the context of the clinical pathway, including its purpose and its intended users (e.g. healthcare professionals, patients, public).	
			SPIRIT-AI 6a (ii) Extension	Describe any pre-existing evidence for the AI intervention.	
	6b	Explanation for choice of comparators			
Objectives	7	Specific objectives or hypotheses			
Trial design	8	Description of trial design including type of trial (eg, parallel group, crossover, factorial, single group), allocation ratio, and framework (eg, superiority, equivalence, non-inferiority, exploratory)			
**Methods: Participants, interventions, and outcomes**					
Study setting	9	Description of study settings (eg, community clinic, academic hospital) and list of countries where data will be collected. Reference to where list of study sites can be obtained	SPIRIT-AI 9 Extension	Describe the onsite and offsite requirements needed to integrate the AI intervention into the trial setting.	
Eligibility criteria	10	Inclusion and exclusion criteria for participants. If applicable, eligibility criteria for study centres and individuals who will perform the interventions (eg, surgeons, psychotherapists)	SPIRIT-AI 10 (i) Elaboration	State the inclusion and exclusion criteria at the level of participants.	
			SPIRIT-AI 10 (ii) Extension	State the inclusion and exclusion criteria at the level of the input data.	
Interventions	11a	Interventions for each group with sufficient detail to allow replication, including how and when they will be administered	SPIRIT-AI 11a (i) Extension	State which version of the AI algorithm will be used.	
			SPIRIT-AI 11a (ii) Extension	Specify the procedure for acquiring and selecting the input data for the AI intervention.	
			SPIRIT-AI 11a (iii) Extension	Specify the procedure for assessing and handling poor quality or unavailable input data.	
			SPIRIT-AI 11a (iv) Extension	Specify whether there is human-AI interaction in the handling of the input data, and what level of expertise is required for users.	
			SPIRIT-AI 11a (v) Extension	Specify the output of the AI intervention.	
			SPIRIT-AI 11a (vi) Extension	Explain the procedure for how the AI intervention’s output will contribute to decision-making or other elements of clinical practice.	
	11b	Criteria for discontinuing or modifying allocated interventions for a given trial participant (eg, drug dose change in response to harms, participant request, or improving/worsening disease)			
	11c	Strategies to improve adherence to intervention protocols, and any procedures for monitoring adherence (eg, drug tablet return, laboratory tests)			
	11d	Relevant concomitant care and interventions that are permitted or prohibited during the trial			
Outcomes	12	Primary, secondary, and other outcomes, including the specific measurement variable (eg, systolic blood pressure), analysis metric (eg, change from baseline, final value, time to event), method of aggregation (eg, median, proportion), and time point for each outcome. Explanation of the clinical relevance of chosen efficacy and harm outcomes is strongly recommended			
Participant timeline	13	Time schedule of enrolment, interventions (including any run-ins and washouts), assessments, and visits for participants. A schematic diagram is highly recommended (see fig 1)			
Sample size	14	Estimated number of participants needed to achieve study objectives and how it was determined, including clinical and statistical assumptions supporting any sample size calculations			
Recruitment	15	Strategies for achieving adequate participant enrolment to reach target sample size			
**Methods: Assignment of interventions (for controlled trials)**					
Sequence generation	16A	Method of generating the allocation sequence (eg, computer-generated random numbers), and list of any factors for stratification. To reduce predictability of a random sequence, details of any planned restriction (eg, blocking) should be provided in a separate document that is unavailable to those who enrol participants or assign interventions			
Allocation concealment mechanism	16b	Mechanism of implementing the allocation sequence (eg, central telephone; sequentially numbered, opaque, sealed envelopes), describing any steps to conceal the sequence until interventions are assigned			
Implementation	16c	Who will generate the allocation sequence, who will enrol participants, and who will assign participants to interventions			
Blinding (masking)	17a	Who will be blinded after assignment to interventions (eg, trial participants, care providers, outcome assessors, data analysts), and how			
	17b	If blinded, circumstances under which unblinding is permissible, and procedure for revealing a participant’s allocated intervention during the trial			
**Methods: Data collection, management, and analysis**					
Data collection methods	18a	Plans for assessment and collection of outcome, baseline, and other trial data, including any related processes to promote data quality (eg, duplicate measurements, training of assessors) and a description of study instruments (eg, questionnaires, laboratory tests) along with their reliability and validity, if known. Reference to where data collection forms can be found, if not in the protocol			
	18b	Plans to promote participant retention and complete follow-up, including list of any outcome data to be collected for participants who discontinue or deviate from intervention protocols			
Data management	19	Plans for data entry, coding, security, and storage, including any related processes to promote data quality (eg, double data entry; range checks for data values). Reference to where details of data management procedures can be found, if not in the protocol			
Statistical methods	20a	Statistical methods for analysing primary and secondary outcomes. Reference to where other details of the statistical analysis plan can be found, if not in the protocol			
	20b	Methods for any additional analyses (eg, subgroup and adjusted analyses)			
	20c	Definition of analysis population relating to protocol non-adherence (eg, as randomised analysis), and any statistical methods to handle missing data (eg, multiple imputation)			
**Methods: Monitoring**					
Data monitoring	21a	Composition of data monitoring committee (DMC); summary of its role and reporting structure; statement of whether it is independent from the sponsor and competing interests; and reference to where further details about its charter can be found, if not in the protocol. Alternatively, an explanation of why a DMC is not needed			
	21b	Description of any interim analyses and stopping guidelines, including who will have access to these interim results and make the final decision to terminate the trial			
Harms	22	Plans for collecting, assessing, reporting, and managing solicited and spontaneously reported adverse events and other unintended effects of trial interventions or trial conduct	SPIRIT-AI 22 Extension	Specify any plans to identify and analyse performance errors. If there are no plans for this, justify why not.	
Auditing	23	Frequency and procedures for auditing trial conduct, if any, and whether the process will be independent from investigators and the sponsor			
**Ethics and dissemination**					
Research ethics approval	24	Plans for seeking research ethics committee/institutional review board (REC/IRB) approval			
Protocol amendments	25	Plans for communicating important protocol modifications (eg, changes to eligibility criteria, outcomes, analyses) to relevant parties (eg, investigators, REC/IRBs, trial participants, trial registries, journals, regulators)			
Consent or ascent	26a	Who will obtain informed consent or assent from potential trial participants or authorised surrogates, and how (see Item 32)			
	26b	Additional consent provisions for collection and use of participant data and biological specimens in ancillary studies, if applicable			
Confidentiality	27	How personal information about potential and enrolled participants will be collected, shared, and maintained in order to protect confidentiality before, during, and after the trial			
Declaration of interests	28	Financial and other competing interests for principal investigators for the overall trial and each study site			
Access to data	29	Statement of who will have access to the final trial dataset, and disclosure of contractual agreements that limit such access for investigators	SPIRIT-AI 29 Extension	State whether and how the AI intervention and/or its code can be accessed, including any restrictions to access or re-use.	
Ancillary and post-trial care	30	Provisions, if any, for ancillary and post-trial care, and for compensation to those who suffer harm from trial participation			
Dissemination policy	31a	Plans for investigators and sponsor to communicate trial results to participants, healthcare professionals, the public, and other relevant groups (eg, via publication, reporting in results databases, or other data sharing arrangements), including any publication restrictions			
	31b	Authorship eligibility guidelines and any intended use of professional writers			
	31c	Plans, if any, for granting public access to the full protocol, participant-level dataset, and statistical code			
**Appendices**					
Informed consent materials	32	Model consent form and other related documentation given to participants and authorised surrogates			
Biological specimens	33	Plans for collection, laboratory evaluation, and storage of biological specimens for genetic or molecular analysis in the current trial and for future use in ancillary studies, if applicable			

* It is strongly recommended that this checklist be read in conjunction with the SPIRIT 2013 Explanation and Elaboration for important clarification on the items.

† Indicates page numbers to be completed by authors during protocol development.

All 15 items included in the SPIRIT-AI Extension passed the threshold of 80% for inclusion at the consensus meeting. SPIRIT-AI 6a (i), SPIRIT-AI 11a (v) and SPIRIT-AI 22 each resulted from the merging of two items after discussion. SPIRIT-AI 11a (iii) did not fulfil the criteria for inclusion based on its initial wording (73% vote to include); however, after extensive discussion and rewording, the consensus group unanimously supported a re-vote at which point it passed the inclusion threshold (97% to include).

### Administrative information

#### SPIRIT-AI 1 (i) Elaboration: Indicate that the intervention involves artificial intelligence/machine learning and specify the type of model.

*Explanation:* Indicating in the protocol title and/or abstract that the intervention involves a form of AI is encouraged, as it immediately identifies the intervention as an artificial intelligence/machine learning intervention, and also serves to facilitate indexing and searching of the trial protocol in bibliographic databases, registries, and other online resources. The title should be understandable by a wide audience; therefore, a broader umbrella term such as “artificial intelligence” or “machine learning” is encouraged. More precise terms should be used in the abstract, rather than the title, unless broadly recognised as being a form of artificial intelligence/machine learning. Specific terminology relating to the model type and architecture should be detailed in the abstract.

#### SPIRIT-AI 1 (ii) Elaboration: State the intended use of the AI intervention.

*Explanation:* The intended use of the AI intervention should be made clear in the protocol’s title and/or abstract. This should describe the purpose of the AI intervention and the disease context.^19^ ^36^ Some AI interventions may have multiple intended uses, or the intended use may evolve over time. Therefore, documenting this allows readers to understand the intended use of the algorithm at the time of the trial.

### Introduction

#### SPIRIT-AI 6a (i) Extension: Explain the intended use of the AI intervention in the context of the clinical pathway, including its purpose and its intended users (such as healthcare professionals, patients, public).

*Explanation:* In order to understand how the AI intervention will fit into a clinical pathway, a detailed description of its role should be included in the protocol background. AI interventions may be designed to interact with different users including healthcare professionals, patients, and the public, and their roles can be wide-ranging (for example, the same AI intervention could theoretically be replacing, augmenting or adjudicating components of clinical decision-making). Clarifying the intended use of the AI intervention and its intended user helps readers understand the purpose for which the AI intervention will be evaluated in the trial.

#### SPIRIT-AI 6a (ii) Extension: Describe any pre-existing evidence for the AI intervention.

*Explanation:* Authors should describe in the protocol any pre-existing published (with supporting references) or unpublished evidence relating to validation of the AI intervention, or lack thereof. Consideration should be given to whether the evidence was for a similar use, setting and target population as the planned trial. This may include previous development of the AI model, internal and external validations, and any modifications made before the trial.

### Participants, interventions, and outcomes

#### SPIRIT-AI 9 Extension: Describe the onsite and offsite requirements needed to integrate the AI intervention into the trial setting.

*Explanation:* There are limitations to the generalisability of AI algorithms, one of which is when they are used outside of their development environment.^37^ ^38^ AI systems are dependent on their operational environment, and the protocol should provide details of the hardware and software requirements to allow technical integration of the AI intervention at each study site. For example, it should be stated if the AI intervention requires vendor-specific devices, if there is a need for specialised computing hardware at each site, or if the sites must support cloud integration, particularly if this is vendor-specific. If any changes to the algorithm are required at each study site as part of the implementation procedure (such as fine-tuning the algorithm on local data), then this process should also be clearly described.

#### SPIRIT-AI 10 (i) Elaboration: State the inclusion and exclusion criteria at the level of participants.

*Explanation:* The inclusion and exclusion criteria should be defined at the participant level as per usual practice in protocols of non-AI interventional trials. This is distinct from the inclusion and exclusion criteria made at the input data level, which is addressed in item 10 (ii).

#### SPIRIT-AI 10 (ii) Extension: State the inclusion and exclusion criteria at the level of the input data.

**Fig 1:**
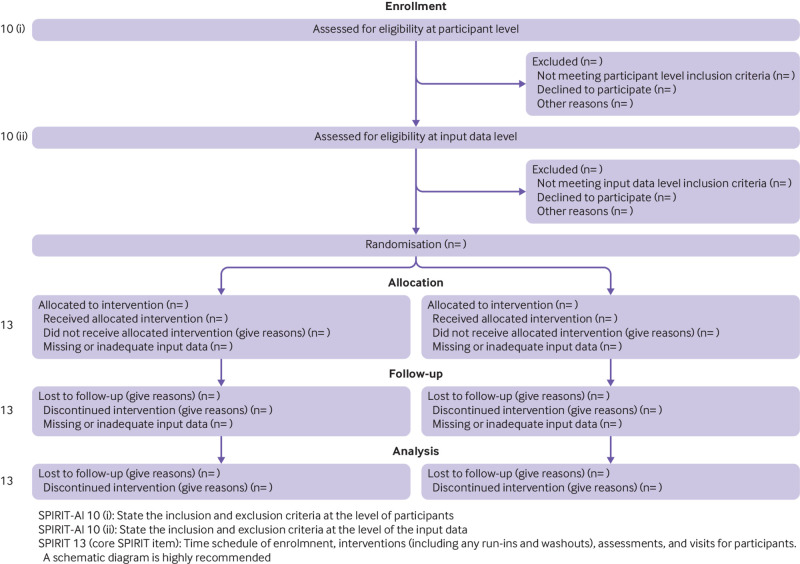
CONSORT 2010 flow diagram - adapted for AI clinical trials

*Explanation:* Input data refer to the data required by the AI intervention to serve its purpose (for example, for a breast cancer diagnostic system, the input data could be the unprocessed or vendor-specific post-processing mammography scan on which a diagnosis is being made; for an early warning system, the input data could be physiological measurements or laboratory results from the electronic health record). The trial protocol should pre-specify if there are minimum requirements for the input data (such as image resolution, quality metrics, or data format), which would determine pre-randomisation eligibility. It should specify when, how, and by whom this will be assessed. For example, if a participant met the eligibility criteria for lying flat for a CT scan as per item 10 (i), but the scan quality was compromised (for any given reason) to such a level that it is no longer fit for use by the AI system, this should be considered as an exclusion criterion at the input data level. Note that where input data are acquired after randomisation (addressed by SPIRIT-20c), any exclusion is considered to be from the analysis, not from enrolment (fig 1).

#### SPIRIT-AI 11a (i) Extension: State which version of the AI algorithm will be used.

*Explanation:* Similar to other forms of software as a medical device, AI systems are likely to undergo multiple iterations and updates in their lifespan. The protocol should state which version of the AI system will be used in the clinical trial, and whether this is the same version that had been used in previous studies to justify the study rationale. If applicable, the protocol should describe what has changed between the relevant versions and the rationale for the changes. Where available, the protocol should include a regulatory marking reference, such as a unique device identifier (UDI) which requires a new identifier for updated versions of the device.^39^

#### SPIRIT-AI 11a (ii) Extension: Specify the procedure for acquiring and selecting the input data for the AI intervention.

*Explanation:* The measured performance of any AI system may be critically dependent on the nature and quality of the input data.^40^ The procedure for how input data will be handled—including data acquisition, selection, and pre-processing before analysis by the AI system—should be provided. Completeness and transparency of this process is integral to feasibility assessment and to future replication of the intervention beyond the clinical trial. It will also help to identify whether input data handling procedures will be standardised across trial sites.

#### SPIRIT-AI 11a (iii) Extension: Specify the procedure for assessing and handling poor quality or unavailable input data.

*Explanation:* As with 10 (ii), input data refer to the data required by the AI intervention to serve its purpose. As noted in item SPIRIT-AI 10 (ii), the performance of AI systems may be compromised as a result of poor quality or missing input data^41^ (for example, excessive movement artefact on an electrocardiogram). The study protocol should specify if and how poor quality or unavailable input data will be identified and handled. The protocol should also specify a minimum standard required for the input data, and the procedure for when the minimum standard is not met (including the impact on, or any changes to, the participant care pathway).

Poor quality or unavailable data can also affect non-AI interventions. For example, suboptimal quality of a scan could impact a radiologist’s ability to interpret it and make a diagnosis. It is therefore important that this information is reported equally for the control intervention, where relevant. If this minimum quality standard is different from the inclusion criteria for input data used to assess eligibility pre-randomisation, this should be stated.

#### SPIRIT-AI 11a (iv) Extension: Specify whether there is human-AI interaction in the handling of the input data, and what level of expertise is required for users.

*Explanation:* A description of the human-AI interface and the requirements for successful interaction when handling input data should be described. Examples include clinician-led selection of regions of interest from a histology slide which is then interpreted by an AI diagnostic system,^42^ or endoscopist selection of a colonoscopy video clip as input data for an algorithm designed to detect polyps.^21^ A description of any planned user training and instructions for how users will handle the input data provides transparency and replicability of trial procedures. Poor clarity on the human-AI interface may lead to a lack of a standard approach and carry ethical implications, particularly in the event of harm.^43^ ^44^ For example, it may become unclear whether an error case occurred due to human deviation from the instructed procedure or if it was an error made by the AI system.

#### SPIRIT-AI 11a (v) Extension: Specify the output of the AI intervention.

*Explanation:* The output of the AI intervention should be clearly defined in the protocol. For example, an AI system may output a diagnostic classification or probability, a recommended action, an alarm alerting to an event, an instigated action in a closed loop system (such as titration of drug infusions), or other. The nature of the AI intervention's output has direct implications on its usability and how it may lead to downstream actions and outcomes.

#### SPIRIT-AI 11a (vi) Extension: Explain the procedure for how the AI intervention’s outputs will contribute to decision-making or other elements of clinical practice.

*Explanation:* Since health outcomes may also critically depend on how humans interact with the AI intervention, the trial protocol should explain how the outputs of the AI system are used to contribute to decision-making or other elements of clinical practice. This should include adequate description of downstream interventions which can impact outcomes. As with SPIRIT-AI 11a (iv), any elements of human-AI interaction on the outputs should be described in detail. Including the level of expertise required to understand the outputs and any training/instructions provided for this purpose. For example, a skin cancer detection system that produces a percentage likelihood as output should be accompanied by an explanation of how this output should be interpreted and acted on by the user, specifying both the intended pathways (such as skin lesion excision if the diagnosis is positive) and the thresholds for entry to these pathways (such as skin lesion excision if the diagnosis is positive and the probability is >80%). The information produced by comparator interventions should be similarly described, alongside an explanation of how such information was used to arrive at clinical decisions for patient management, where relevant.

### Monitoring

#### SPIRIT-AI 22 Extension: Specify any plans to identify and analyse performance errors. If there are no plans for this, explain why not.

*Explanation:* Reporting performance errors and failure case analysis is especially important for AI interventions. AI systems can make errors which may be hard to foresee but which, if allowed to be deployed at scale, could have catastrophic consequences.^45^ Therefore, identifying cases of error and defining risk mitigation strategies are important for informing when the intervention can be safely implemented and for which populations. The protocol should specify whether there are any plans to analyse performance errors. If there are no plans for this, a justification should be included in the protocol.

### Ethics and dissemination

#### SPIRIT-AI 29 Extension: State whether and how the AI intervention and/or its code can be accessed, including any restrictions to access or re-use.

*Explanation:* The protocol should make clear whether and how the AI intervention and/or its code can be accessed or re-used. This should include details regarding the license and any restrictions to access.

## Discussion

The SPIRIT-AI extension provides international consensus-based guidance on AI-specific information that should be reported in clinical trial protocols alongside SPIRIT 2013 and other relevant SPIRIT extensions.^4^ ^46^ It comprises 15 items: three elaborations to the existing SPIRIT 2013 guidance in the context of AI trials and 12 new extensions. The guidance does not aim to be prescriptive regarding the methodological approach to AI trials; rather it aims to promote transparency in reporting the design and methods of a clinical trial to facilitate understanding, interpretation, and peer review.

A number of extension items relate to the intervention (items 11(i-vi)), its setting (item 9), and intended role (item 6a (i)). Specific recommendations were made pertinent to AI systems relating to algorithm version, input and output data, integration into trial settings, expertise of the users, and protocol for acting on the AI system’s recommendations. It was agreed that these details are critical for independent evaluation of the study protocol. Journal editors reported that, despite the importance of these items, they are currently often missing from trial protocols and reports at the time of submission for publication, providing further weight to their inclusion as specifically listed extension items.

A recurrent focus of the Delphi comments and consensus group discussion was around safety of AI systems. This is in recognition that these systems, unlike other health interventions, can unpredictably yield errors which are not easily detectable or explainable by human judgment. For example, changes to medical imaging which are invisible, or appear random, to the human eye may change the likelihood of the resultant diagnostic output entirely.^47^ ^48^ The concern is, given the theoretical ease at which AI systems could be deployed at scale, any unintended harmful consequences could be catastrophic. Two extension items were added to address this. SPIRIT-AI item 6a (ii) requires specification of the prior level of evidence for validation of the AI intervention. SPIRIT-AI item 22 requires specification of any plans to analyse performance errors, to emphasise the importance of anticipating systematic errors made by the algorithm and their consequences.

One topic which was raised in the Delphi survey responses and consensus meeting, which is not included in the final guidelines, is “continuously evolving” AI systems (also known as “continuously adapting” or “continuously learning”). These are AI systems with the ability to continuously train on new data, which may cause changes in performance over time. The group noted that, while of interest, this field is relatively early in its development without tangible examples in healthcare applications, and that it would not be appropriate for it to be addressed by SPIRIT-AI at this stage.^49^ This topic will be monitored and revisited in future iterations of SPIRIT-AI. It is worth noting that incremental software changes, whether continuous or iterative, intentional or unintentional, could have serious consequences on safety performance after deployment. It is therefore of vital importance that such changes are documented and identified by software version and a robust post-deployment surveillance plan is in place.

This study is set in the current context of AI in health; therefore, several limitations should be noted. First, at the time of SPIRIT-AI development there were only seven published trials and no published trial protocols in the field of AI for healthcare. Thus, the discussion and decisions made during the development of SPIRIT-AI are not always supported by existing real-world examples. This arises from our stated aim to address the issues of poor protocol development in this field as early as possible, recognising the strong drivers in the field and the specific challenges of study design and reporting for AI. As the science and study of AI evolves, we welcome collaboration with investigators to co-evolve these reporting standards to ensure their continued relevance. Second, the literature search of AI RCTs used terminology such as “artificial intelligence,” “machine learning,” and “deep learning” but not terms such as “clinical decision support systems” and “expert systems,” which were more commonly used in the 1990s for technologies underpinned by AI systems and share similar risks with recent examples.^50^ It is likely that such systems, if published today, would be indexed under “AI” or “machine learning”; however, clinical decision support systems were not actively discussed during this consensus process. Third, the initial candidate items list was generated by a relatively small group of experts consisting of steering group members and additional international experts. However, additional items from the wider Delphi group were taken forward for consideration by the consensus group, and no new items were suggested during the consensus meeting or post-meeting evaluation.

As with the SPIRIT statement, the SPIRIT-AI extension is intended as a minimum reporting guidance, and there are additional AI-specific considerations for trial protocols which may warrant consideration (see appendix, page 2: supplementary table 2). This extension is particularly aimed at investigators planning or conducting clinical trials; however, it may also serve as useful guidance for developers of AI interventions in earlier validation stages of an AI system. Investigators seeking to report studies developing and validating the diagnostic and predictive properties of AI models should refer to TRIPOD-ML (Transparent Reporting of a Multivariable Prediction Model for Individual Prognosis or Diagnosis - Machine Learning)^24^ and STARD-AI (Standards for Reporting Diagnostic accuracy studies - Artificial Intelligence),^51^ both of which are currently under development. Other potentially relevant guidelines are registered with the EQUATOR network which are agnostic to study design.^52^ The SPIRIT-AI extension is expected to encourage careful early planning of AI interventions for clinical trials, and this, in conjunction with CONSORT-AI, should help to improve the quality of trials for AI interventions.

There is widespread recognition that AI is a rapidly evolving field and there will be the need to update SPIRIT-AI as the technology, and newer applications for it, develop. Currently, most applications of AI/ML involve disease detection, diagnosis, and triage, and this is likely to have influenced the nature and prioritisation of items within SPIRIT-AI. As wider applications that utilise “AI as therapy” emerge, it will be important to re-evaluate SPIRIT-AI in the light of such studies. Additionally, advances in computational techniques and the ability to integrate them into clinical workflows will bring new opportunities for innovation that benefits patients. However, they may be accompanied by new challenges of study design and reporting to ensure transparency, minimise potential biases and ensure that the findings of such a study are trustworthy and the extent to which they may be generalisable. The SPIRIT-AI and CONSORT-AI Steering Group will continue to monitor the need for updates.

## Web Extra

Extra material supplied by the author

Appendix: Supplementary table 1 (details of Delphi survey and consensus meeting participants) and table 2 (details of Delphi survey and consensus meeting decisions)

Supplementary fig 1: Decision tree for inclusion/exclusion and extension/elaboration

Supplementary fig 2: Checklist development process

## Notes

**The SPIRIT-AI and CONSORT-AI Working Group:** Samantha Cruz Rivera,^1,2^ Xiaoxuan Liu,^2,3,4,5,6^ An-Wen Chan,^7^ Alastair K Denniston,^1,2,3,4,5,8^ Melanie J Calvert,^1,2,6,9,10,11^ Hutan Ashrafian,^12,13^ Andrew L Beam,^14^ Gary S Collins,^15^ Ara Darzi,^12,13^ Jonathan J Deeks,^10,16^ M Khair ElZarrad,^17^ Cyrus Espinoza,^18^ Andre Esteva,^19^ Livia Faes,^4,20^ Lavinia Ferrante di Ruffano,^12^ John Fletcher,^21^ Robert Golub,^22^ Hugh Harvey,^23^ Charlotte Haug,^24^ Christopher Holmes,^25,26^ Adrian Jonas,^27^ Pearse A Keane,^8^ Christopher J Kelly,^28^ Aaron Y Lee,^29^ Cecilia S Lee,^29^ Elaine Manna,^18^ James Matcham,^30^ Melissa McCradden,^31^ David Moher,^32,33^ Joao Monteiro,^34^ Cynthia Mulrow,^35^ Luke Oakden-Rayner,^36^ Dina Paltoo,^37^ Maria Beatrice Panico,^38^ Gary Price,^18^ Samuel Rowley,^39^ Richard Savage,^40^ Rupa Sarkar,^41^ Sebastian J Vollmer,^26,42^ Christopher Yau,^26,43^

1. Centre for Patient Reported Outcome Research, Institute of Applied Health Research, University of Birmingham, Birmingham, UK.

2. Birmingham Health Partners Centre for Regulatory Science and Innovation, University of Birmingham, Birmingham, UK

3. Academic Unit of Ophthalmology, Institute of Inflammation and Ageing, College of Medical and Dental Sciences, University of Birmingham, UK

4. Department of Ophthalmology, University Hospitals Birmingham NHS Foundation Trust, Birmingham, UK

5. Moorfields Eye Hospital NHS Foundation Trust, London, UK

6. Health Data Research UK, London, UK

7. Department of Medicine, Women’s College Research Institute, Women’s College Hospital, University of Toronto, Ontario, Canada

8. National Institute of Health Research Biomedical Research Centre for Ophthalmology, Moorfields Hospital London NHS Foundation Trust and University College London, Institute of Ophthalmology, London, UK

9. National Institute of Health Research Surgical Reconstruction and Microbiology Centre, University of Birmingham and University Hospitals Birmingham NHS Foundation Trust, Birmingham, UK

10. National Institute of Health Research Birmingham Biomedical Research Centre, University of Birmingham and University Hospitals Birmingham NHS Foundation Trust, Birmingham, UK

11. National Institute of Health Research Applied Research Collaborative West Midlands, Birmingham, UK

12. Institute of Global Health Innovation, Imperial College London, London, UK

13. Patient Safety Translational Research Centre, Imperial College London, London, UK

14. Harvard T.H. Chan School of Public Health, Boston, MA, USA

15. Centre for Statistics in Medicine, University of Oxford, Oxford, UK

16. Institute of Applied Health Research, University of Birmingham, Birmingham, UK

17. Food and Drug Administration, Maryland, USA

18. Patient Representative

19. Salesforce Research, San Francisco, CA, USA

20. Department of Ophthalmology, Cantonal Hospital Lucerne, Lucerne, Switzerland

21. *The BMJ*, London, UK

22. *JAMA (Journal of the American Medical Association)*, Chicago, IL, USA

23. Hardian Health, London, UK

24. *New England Journal of Medicine*, Massachusetts, USA

25. Department of Statistics and Nuffield Department of Medicine, University of Oxford, Oxford, UK

26. Alan Turing Institute, London, UK

27. The National Institute for Health and Care Excellence (NICE), London, UK

28. Google Health, London, UK

29. Department of Ophthalmology, University of Washington, Seattle, Washington, USA

30. AstraZeneca Ltd, Cambridge, UK

31. The Hospital for Sick Children, Toronto, Canada

32. Centre for Journalology, Clinical Epidemiology Program, Ottawa Hospital Research Institute, Ottawa, Canada

33. School of Epidemiology and Public Health, University of Ottawa, Ottawa, Canada

34. *Nature Research*, New York, NY, USA

35. *Annals of Internal Medicine*, Philadelphia, PA, USA

36. Australian Institute for Machine Learning, North Terrace, Adelaide, Australia

37. National Institutes of Health, Maryland, USA

38. Medicines and Healthcare products Regulatory Agency, London, UK

39. Medical Research Council, London, UK

40. PinPoint Data Science, Leeds, UK

41. The Lancet Group, London, UK

42. University of Warwick, Coventry, UK

43. University of Manchester, Manchester, UK

**Data availability:** Data requests should be made to the corresponding author and release will be subject to consideration by the SPIRIT-AI and CONSORT-AI Steering Group.
