# Supplementary material for: Guidelines for clinical trial protocols for interventions involving artificial intelligence: the SPIRIT-AI Extension
Source: BMJ. 2020 Sep 9;370:m3210. doi: 10.1136/bmj.m3210 (PMC7490785; doi:10.1136/bmj.m3210)
Supplement: Supplementary file 2 — Supplementary fig 1: Decision tree for inclusion/exclusion and extension/elaboration [file crus059982.wf1.pdf]

Supplementary Figure 1 (SPIRIT-AI): decision tree for inclusion/exclusion and extension/elaboration.

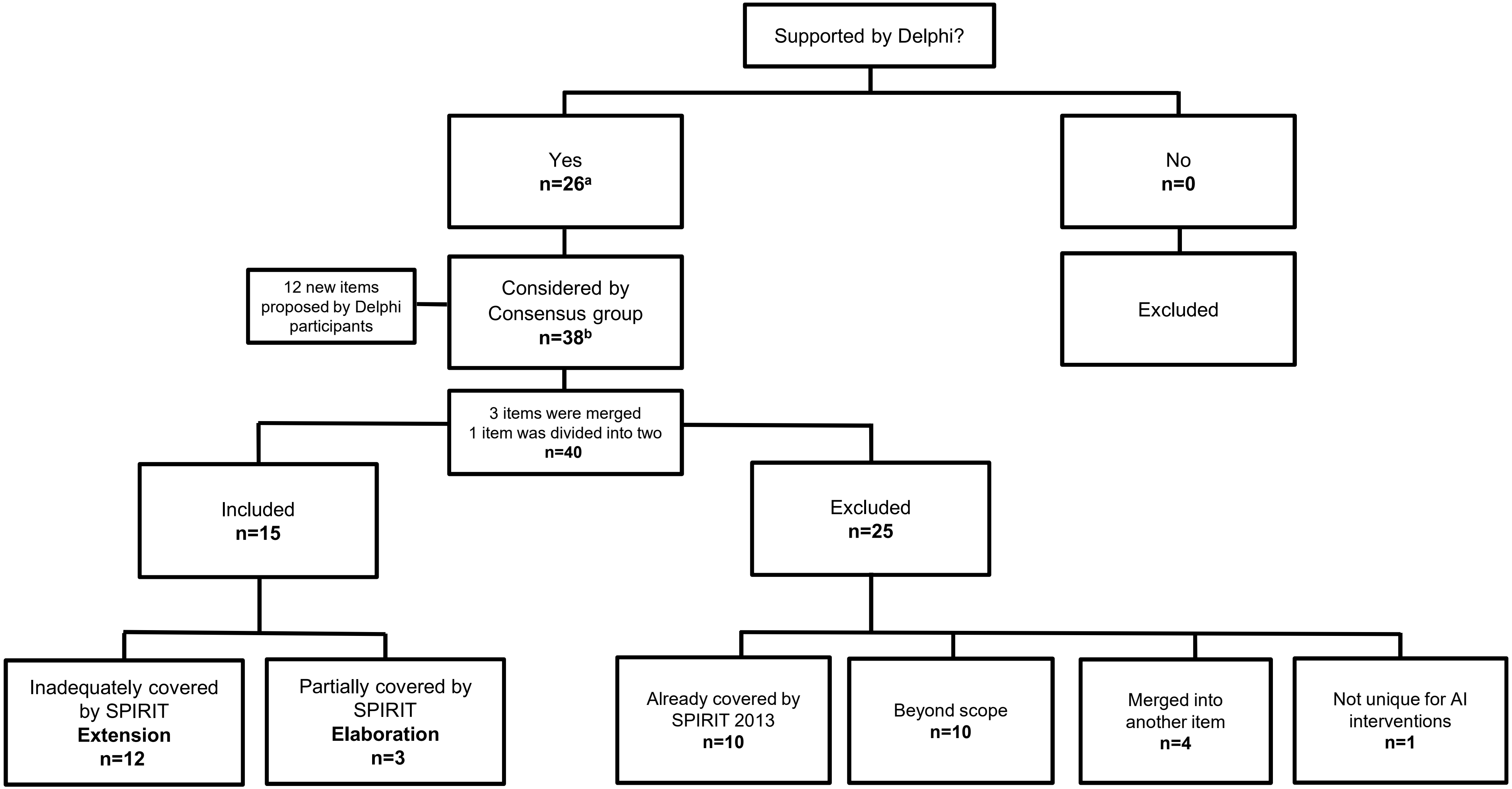

[a] Delphi exercise: inclusion criteria threshold, median score (IQR) ≥4 for (1-3) not important, (4-6) important but not critical and (7-9) important and critical items.
[b] Consensus meeting: inclusion criteria threshold, ≥80% voted included.
