# Supplementary figures and images for: Guidelines for clinical trial protocols for interventions involving artificial intelligence: the SPIRIT-AI Extension
Source: BMJ. 2020 Sep 9;370:m3210. doi: 10.1136/bmj.m3210 (PMC7490785; doi:10.1136/bmj.m3210)

**Supplementary Figure 2: Checklist Development Process**

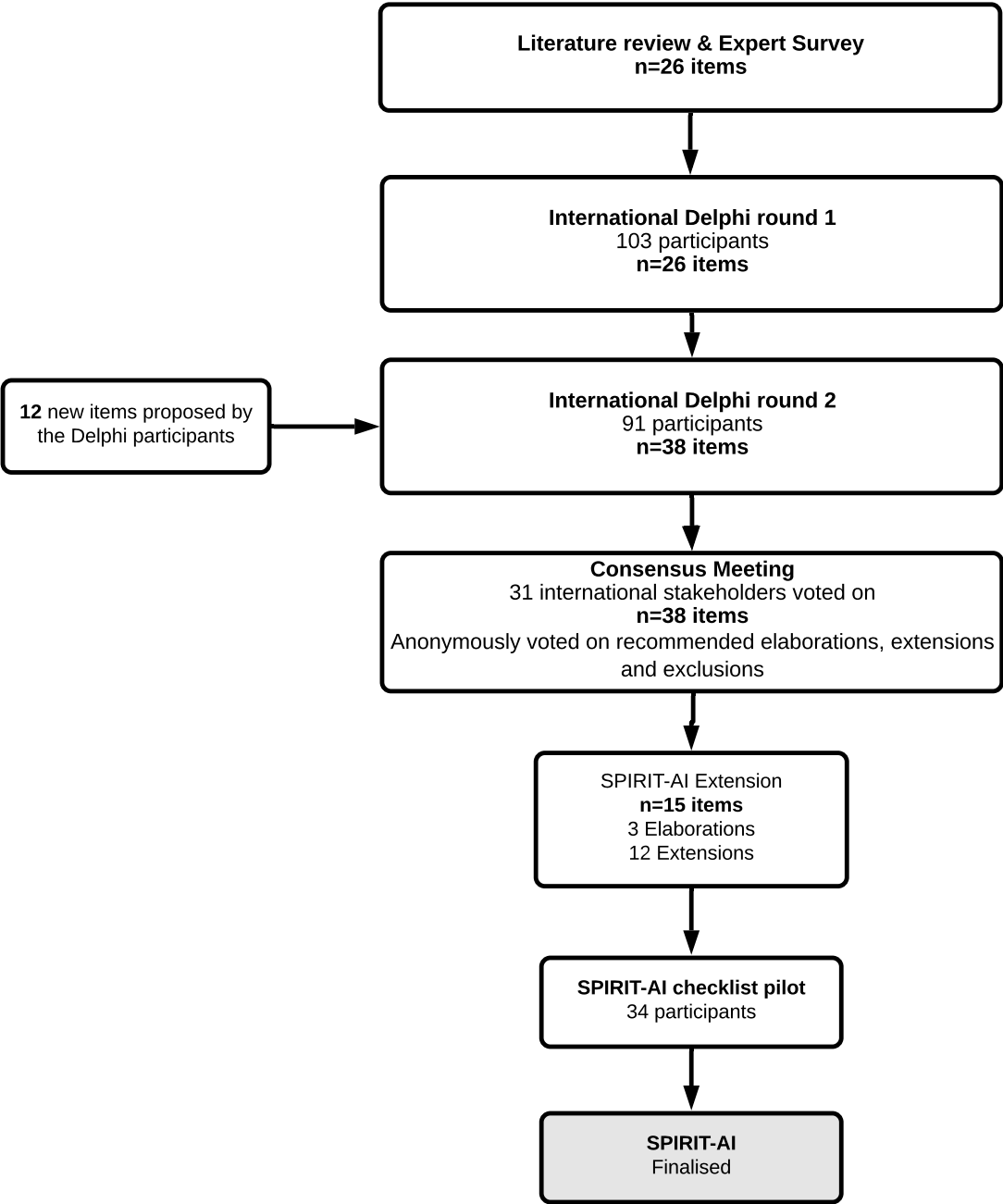

Supplement: Supplementary file 3 — Supplementary fig 2: Checklist development process [file crus059982.wf2.pdf]
